# Morphological changes of TMJ disc in surgically treated ADDwoR patients: a retrospective study

**DOI:** 10.1186/s12903-022-02469-8

**Published:** 2022-10-01

**Authors:** Ruiyu Wang, Ruiye Bi, Yao Liu, Pinyin Cao, Bassam Abotaleb, Songsong Zhu

**Affiliations:** State Key Laboratory of Oral Diseases, National Clinical Research Center for Oral Diseases, Department of Orthognathic and TMJ Surgery, West China Hospital of Stomatology, Sichuan University, No. 14, 3rd Section of Ren Min Nan Road, Chengdu, 610041 China

**Keywords:** Temporomandibular joint disc, Magnetic resonance imaging, Disc repositioning surgery, Three-dimensional model

## Abstract

**Background:**

This study aimed to quantify the morphological changes of temporomandibular joint (TMJ) discs after disc repositioning surgery using the three-dimensional (3D) modeling.

**Methods:**

Thirty patients who diagnosed with unilateral ADDwoR were included to compare the morphological differences between ADDWoR discs and normal discs, and fifteen patients who experienced unilateral or bilateral disc repositioning surgery were included to analyze the morphological changes before and after disc repositioning surgery. Disc 3D reconstruction and analyses were performed using magnetic resonance imaging (MRI) data.

**Results:**

In the unilateral ADDwoR patients, volume, superficial area, length, and maximum longitudinal-sectional area of the ADDwoR disc were significantly smaller compared with the non-affected discs. However, there was no significant difference in width and cross-sectional areas between ADDwoR discs and non-affected discs. In patients who subjected to disc repositioning surgery, disc volume, superficial area, length, width and maximum longitudinal-sectional area of TMJ discs were markedly increased 6 months after surgery.

**Conclusions:**

This study demonstrated that the TMJ discs tended to be morphologically smaller in volume and shorter in length under ADDwoR status. Importantly, the ADDwoR discs tended to morphologically recover toward non-affected discs after 6 months follow-up following TMJ disc repositioning surgery.

**Supplementary Information:**

The online version contains supplementary material available at 10.1186/s12903-022-02469-8.

## Introduction

Anterior disc displacement with and without reduction (ADDwR and ADDwoR) are the most common types of temporomandibular joint disorders (TMD), affecting up to 25% of the population [1–3]. In general, ADDwoR refers to the late stage of ADDwR, which is characterized by the disc being displaced anteriorly when the mouth is closed and not being able to reduce to normal position with mouth opening. In ADDwoR patients, the main clinical symptoms include TMJ noises, limitation of mouth opening and joint pain. Furthermore, TMJ osteoarthritis and condylar resorption can be noticed as the illness development [4].

It has been found that with the development of ADDwoR, not only the condyle becomes subjected to bone resorption, but also the TMJ disc experience degeneration. The disc can manifest both pathological and morphological changes. Previous studies reported that pathological disc vascularization would occur in the posterior band of the disc during ADDwoR resulting in disc morphological changes, such as perforation [5, 6]. Moreover, imaging studies showed that greater deformations of the disc including folding and shortened configuration, were more prevalent in the ADDwoR patients compared to the ADDwR patients [7]. These findings imply that the disc morphological changes continued to be worsen if not received appropriate clinical interventions.

Disc repositioning surgery has been widely used in the last decades in the treatment of ADDwoR patients [8–11]. This surgical approach significantly reduced TMJ noises, improved mouth-opening and relieved TMJ pain for symptomatic patients [12]. In terms of the imaging evaluation of the surgery outcome, most of the previous studies paid attention to the disc-condyle relationship, and disc recapture was observed in the majority of patients [8, 13, 14]. In addition, Liu et al. [15] found that the length of TMJ disc became significantly longer after successful repositioning surgery. However, comprehensive 3D quantitative analysis of the morphological changes of TMJ disc in patients with ADDwoR still required to enable the clinicians to evaluating surgical effect after TMJ disc repositioning surgery.

The study aimed to investigate morphological differences of the TMJ disc in three dimensions between the ADDwoR side and the normal side in unilateral ADDwoR patients. This study also assessed three-dimensional morphological improvement of the disc following disc repositioning surgery.

## Materials and methods

### Patients

This study included cross-sectional observation of patients who met the following inclusion criteria for analyzing disc morphological differences between the ADDwoR discs and the normal discs: (1) Patients diagnosed as unilateral ADDwoR based on MRI according to Diagnostic Criteria for Temporomandibular Disorders (DC/TMD); (2) In the meantime the other side was observed with no ADDwoR, ADDwR or other significant image abnormalities (This side was regarded as non-affected side). The study furtherly included patients who met the following inclusion criteria for analyzing disc morphological improvement after the surgery: (1) Diagnosed with unilateral or bilateral ADDwoR based on MRI according to DC/TMD; (2) Subjected for disc repositioning surgery with follow-up of at least 6 months. Patients with systemic arthritis, TMJ trauma or history of TMJ therapy were excluded. The study was approved by the institutional review board of West China Hospital of Stomatology, Sichuan University and all patients signed the informed consent.

### TMJ repositioning surgical procedures

Patients diagnosed with ADDwoR were subjected to disc repositioning surgery under general anesthesia. The procedures involved has been described in previous study of Goncalves et al. [14]. Briefly, an endaural incision with inverted L extension was made extending from the hairline to the level where the ear lobe meets the tragus [3]. Then a blunt dissection was done until the inferior of zygomatic arch and capsule of TMJ were exposed. An incision which follows the shape of the glenoid fossa was carried through the TMJ capsule to expose the upper compartment above the TMJ disc. Followed by releasing the anterior attachment of the disc to be passively relocate in the correct position. Mitek anchor was then fixed into the posterolateral aspect of the condyle neck, and linked two sutures of the anchor were then passed through the medial and lateral aspects of the posterior band of the disc to suture back to the anchor. The final stable position of the disc should be located above the condyle precisely with slight overcorrection. When the disc-condyle relationship remained harmonious after gently moving the condyle in various directions, the procedure was judged successful. After disc repositioning surgery, all patients received postoperative splint therapy for 6 months.

### MRI examinations

A 1.5 T imager (Signa; General Electric, Milwaukee, WI) with a TMJ coil was used for imaging the TMJ in all subjects. PD weighted and T2 weighted image sequences were employed for image acquisition. A sagittal scan was set to be parallel to the short axis of the condylar head, and a coronal scan was set to be parallel to the long axis of the condylar head [16]. Parameters used in our department for TMJ imaging were as follows: PD weighted images; TR 2300 ms, TE 47 ms, and T2 weighted images; TR 2500 ms, TE 47 ms, matrix size 288 × 256, FOV 140 × 126 mm, slice thickness 2 mm, slice intervals 0.1 mm, and scan time 4.5 min. MRI was taken when mouth was open (maximum mouth opening position) and closed (maximum intercuspation).

To compare the disc morphological differences between ADDWoR side and normal side, Digital Imaging and Communications in Medicine (DICOM) at the first visit was included. Furthermore, for analyzing disc morphological changes between preoperation and postoperation, DICOM was acquired at the first visit and at 6 months after surgery.

In MRI, a normal disc was defined as one located over the condyle in a position that the middle zone of the disc was articulated against the anterior prominence of the condyle. An ADDwoR disc was defined as one observed in an anterior displaced position when the mouth was closed, and the posterior band remained misplaced in relation to the anterior surface of the condyle during mouth opening.

### TMJ disc morphological measurement

**Fig. 1:**
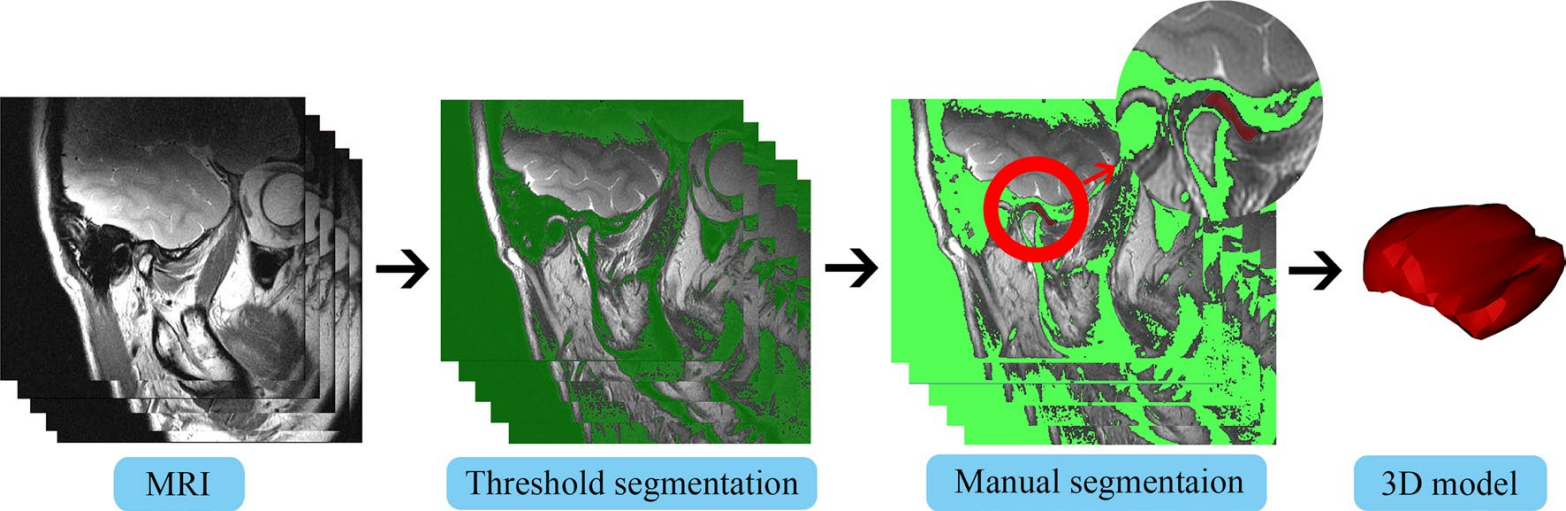
The workflow of the 3D reconstruction of the TMJ disc

DICOM of the MRI in the closed mouth position were used for threshold segmentation (Greyscale Value: 0–235) using Mimics Medical 21.0 software (Materialise, Belgium). Owing to the similar density between TMJ disc, the bone cortex of the condyle, articular eminence and the fossa, an experienced assessor would revise the segmentation manually in order to amend the disc border. After isolating the disc mask from the image background, the masks of discs were acquired to create 3D models of the discs (Fig. 1; Additional file 3).

The overall disc volume and dimension of superficial area of the 3D disc model were then calculated. Disc length was measured as the maximum length along the sagittal plane, while the width was measured as maximum length along coronal plane. Furthermore, the maximum cross-sectional area in coronal plane and maximum longitudinal-sectional area in sagittal plane was measured. The condylar height was defined as the vertical distance between lines N and N′ as previously described [17]. The tangent of the posterior margin of mandibular ramus and the condyle was defined by Line R. Line N was a vertical line of the line R and tangent to the lowest point of the mandibular sigmoid notch. Line N' was designated as a parallel to line N and tangent to the condylar head (Additional file 4).

### Statistical analysis

The SPSS software package version 21.0 was used to analyze the morphological differences between the affected side and the non-affected side, and the morphological changes between preoperation and postoperation in disc volume, superficial area, disc length and width, maximum cross-sectional area and longitudinal-sectional area. D'Agostino-Pearson test was applied to normality assessment using Graphpad prism 8.3.0. Comparisons were made using the paired t-test. Pearson’s correlation analysis was used to investigate the correlation between the condyle height and the morphological differences between the affected side and the non-affected side in the ADDwoR patients. Inter and intra observer data reliability was calculated using intraclass correlation coefficient (ICC). A *P* value < 0.05 is considered as statistically significant.

## Results

Thirty patients diagnosed with unilateral ADDwoR were included in this study to investigate the three-dimensional morphological differences of the TMJ disc between the affected side and the non-affected side, with an average age of 27.6 years old. Fifteen patients who diagnosed with ADDwoR and experienced disc repositioning surgeries were included for analyzing morphological changes of TMJ disc preoperatively and 6 months after the repositioning surgery, with an average age of 25.8 years old. Five patients experienced both sides surgery of TMJ, and ten patients experienced single side surgery of TMJ. Patient’s demographic and clinical characteristics were shown in Table 1. Data distributions in every group accorded with normal distribution using D'Agostino-Pearson test. Inter and intra observer data reliability measurements were applied at a 95% confidence interval with ICC was more than 0.85.

**Table 1:** The basic information of patients

Study variables	Patients with unilateral ADDwoR	Patients following the surgery
No. of patients		
Male	3	2
Female	27	13
Age (year)		
Range	11–61	18–61
Mean	27.6	25.8
TMJ status		
ADDwoR	30	20
Non-affected side	30	10*

*The non-affected TMJs without any surgical interventions were not included in this study

### Morphological differences of TMJ discs in unilateral ADDwoR patients

**Fig. 2:**
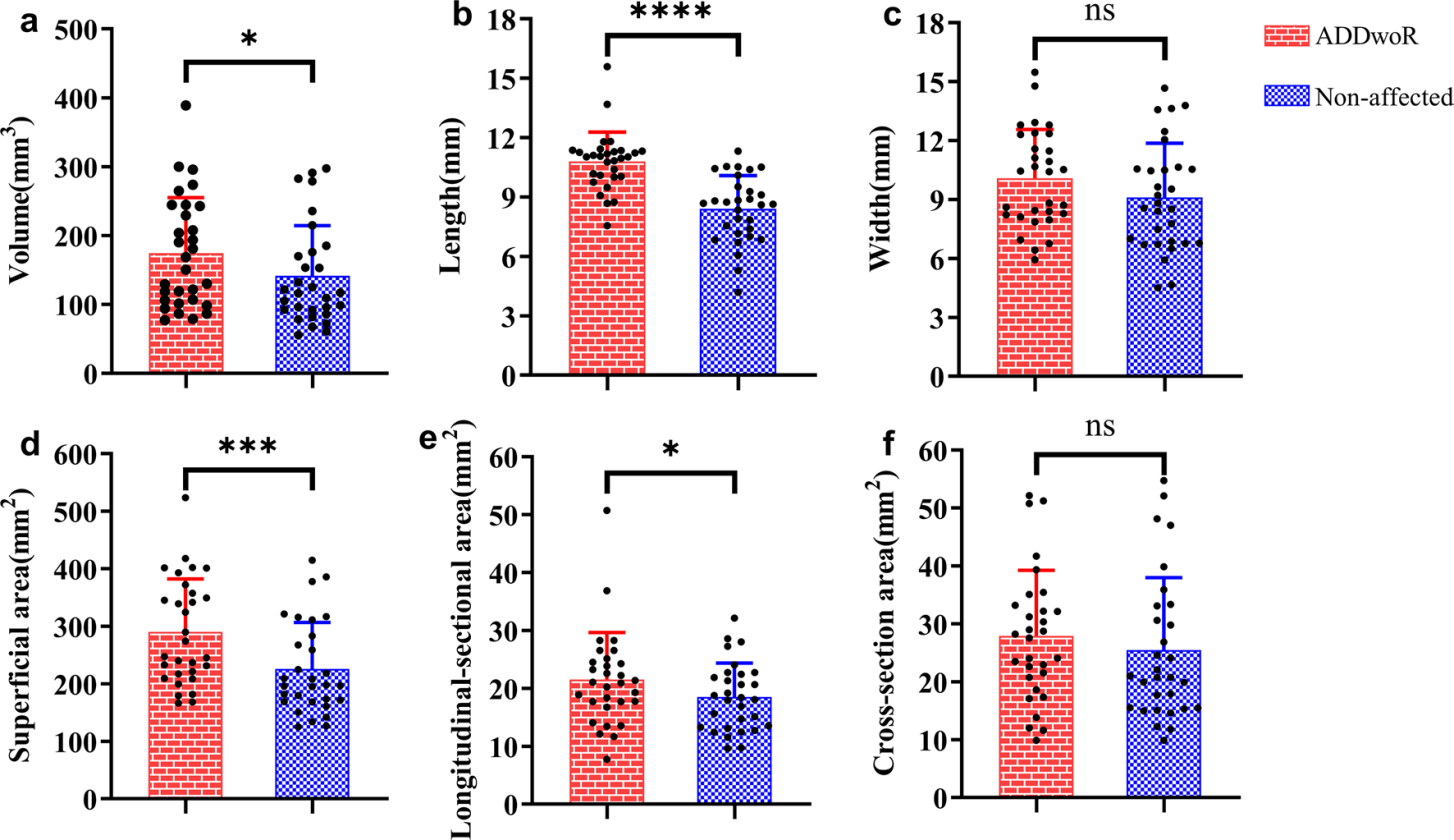
Morphological differences between non-affected side and ADDwoR side. * = *P* < 0.05, ** = *P* < 0.01, *** = *P* < 0.001, **** = *P* < 0.0001, ns = *P* > 0.05. non-affected = non affected side of TMJ. ADDwoR = anterior disc displacement without reduction side. **a** Volume difference between non-affected side and ADDwoR. **b** Length difference between non-affected side and ADDwoR. **c** Width difference between non-affected side and ADDwoR. **d** Superficial area difference between non-affected side and ADDwoR. **e** Longitudinal-sectional area difference between non-affected side and ADDwoR. **f** Cross-section area difference between non-affected side and ADDwoR

The mean of disc volume on the affected side (141.4 ± 72.8mm^3^) was significantly smaller than that of the non-affected side (174.5 ± 80.7mm^3^; *P* = 0.0173). At the same time, the mean of superficial area of the affected side disc was significantly smaller than that of the non-affected side (226.0 ± 80.7 mm^2^ vs. 290.6 ± 91.8 mm^2^, (*P* = 0.0006). In the sagittal plane, the longitudinal-sectional area of the ADDwoR side was less than that in the non-affected side (18.5 ± 5.9 mm^2^ vs. 21.5 ± 8.1 mm^2^; *P* = 0.0112), and the length of ADDwoR discs were significantly less than that on non-affected discs (8.4 ± 1.7 mm vs. 10.8 ± 1.5 mm, *P* < 0.0001). Furthermore, there was no significant difference in width and cross-sectional areas between ADDwoR discs and non-affected discs (Width: 9.1 ± 2.6 mm vs. 10.1 ± 2.5 mm, *P* = 0.0957;Cross-sectional areas: 25.5 ± 12.5 mm^2^ vs. 27.9 ± 11.3 mm^2^, *P* = 0.2454; Fig. 2; Additional file 1).

### Morphological changes of TMJ discs following repositioning surgery

**Fig. 3:**
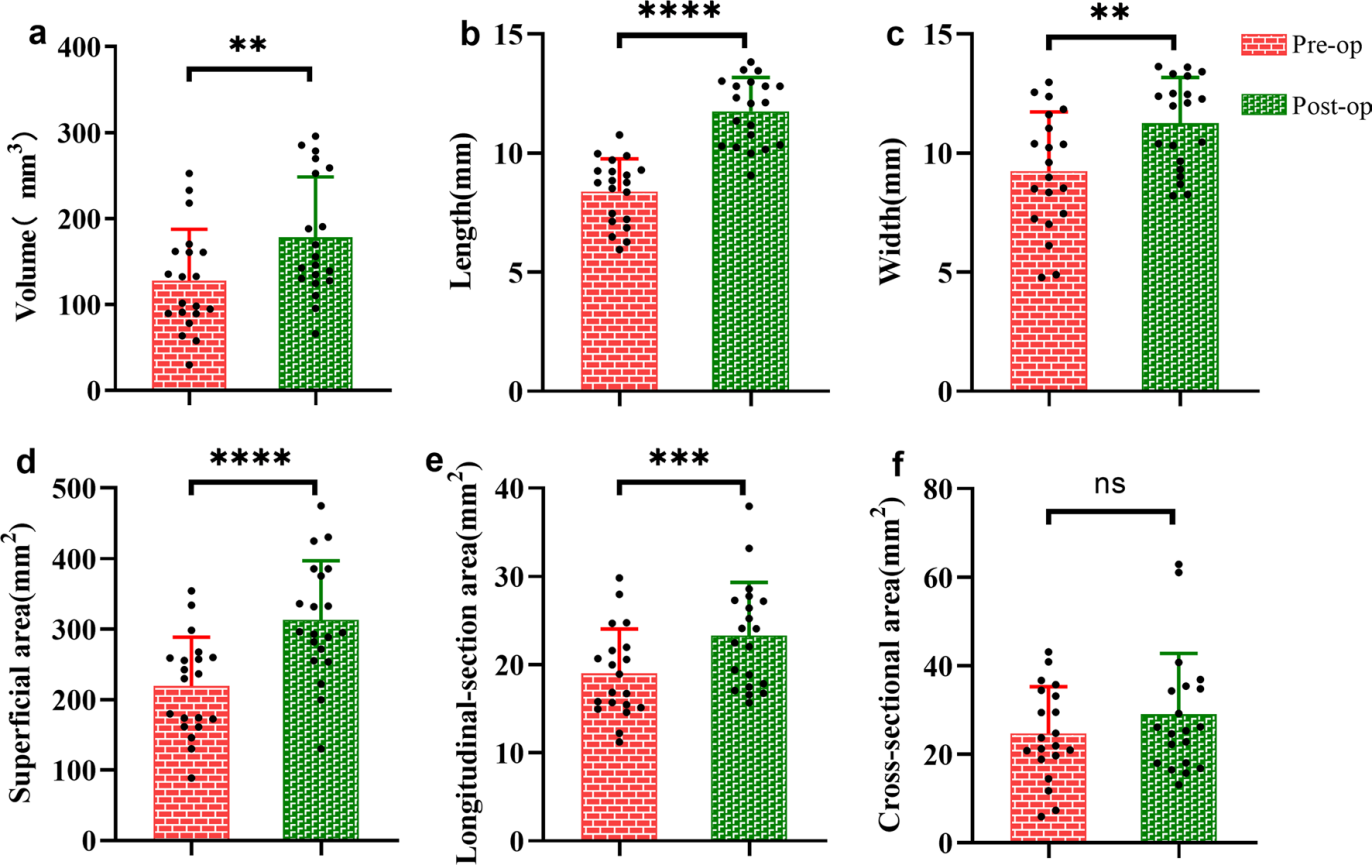
Morphological changes after disc repositioning surgery. pre-op = preoperation. post-op = postoperation. * = *P* < 0.05, ** = *P* < 0.01, *** = *P* < 0.001, **** = *P* < 0.0001, ns = *P* > 0.05. **a** Volume changes after disc repositioning surgery. **b** Length changes after disc repositioning surgery. **c** Width changes after disc repositioning surgery. **d** Superficial area changes after disc repositioning surgery. **e** Longitudinal-section area changes after disc repositioning surgery. **f** Cross-sectional area changes after disc repositioning surgery

After surgery, the volume of the repositioned discs was significantly increased compared to that before the surgical treatment (178.0 ± 70.5mm^3^ vs. 127.5 ± 59.8mm^3^, *P* = 0.0050). There was also an increase of the superficial area from 219.1 ± 69.5 to 313.1 ± 84.0 mm^2^ (*P* < 0.0001). In the sagittal plane, the longitudinal-sectional area increased from 19.0 ± 5.1 to 23.3 ± 6.0 mm^2^ (*P* = 0.0003). In the coronal plane, the cross-sectional area increased from 24.7 ± 10.5 to 29.0 ± 13.7 mm^2^, but this increase was not statistically significant (*P* = 0.1677). Regarding the length and width of the discs, the length of disc significantly increased from 8.4 ± 1.4 to 11.8 ± 1.4 mm, and the disc width increased from 9.2 ± 2.5 to 11.3 ± 1.9 mm, (*P* < 0.0001, *P* = 0.0035, respectively; Fig. 3; Additional file 2).

### Correlation between the disc morphological changes and condyle height

Statistically significant correlations existed between the decrease of the condyle height and the loss of volume, superficial area, length, longitudinal-sectional area and width in patients with unilateral ADDwoR. However, cross-sectional area, were not significantly correlated with condyle height (*P* > 0.05). A greater decrease of the condyle height correlated with a higher reduction of volume, superficial area, length, longitudinal-sectional area and width (*P* = 0.0023, *P* = 0.0028, *P* = 0.0104, *P* = 0.0327, *P* = 0.097, respectively; Table 2).

**Table 2:** The correlation between the disc morphology and condylar height

Disc morphology	r	*P*
Volume	0.5344	0.0023**
Superficial area	0.5246	0.0028**
Length	0.4608	0.0104*
Longitudinal-section area	0.3908	0.0327*
Width	0.4644	0.0097**
Cross-sectional area	0.2170	0.2494

*P < 0.05; **P < 0.01

## Discussion

This study aimed to quantify the morphological changes of temporomandibular joint (TMJ) discs after disc repositioning surgery using the three-dimensional modeling. Although the disc repositioning surgery has been demonstrated effective at alleviating symptoms and restoring disc-condyle relationship in previous studies [18, 19], there was very limited knowledge about the clinical outcomes of the TMJ disc itself after surgical treatment. Therefore, evaluation of the morphological changes of TMJ discs following surgical treatment was awaiting implemented for better assessing the surgical effect in ADDwoR patients.

First, we performed a comparison of disc morphological difference between the affected side and the non-affected side in patients with unilateral ADDwoR. Significant differences were found when disc volume and the superficial area were compared between the ADDwoR side and the non-affected side. A previous study observed that TMJ disc volume could change with negative pressure of TMJ cavity by means of absorbing or squeezing liquid [20]. In our study, however, the volumes of TMJ discs were measured in closed mouth position, therefore the pressure change may not be the principal reason for the volume decrease. In addition to the pressure change, the volume difference of TMJ disc could be accounted for physiological or pathological remodeling in some situations according to several animal studies [21, 22], while researchers in these studies observed that the intermediate zone and posterior band were thicker in the experimental group with a non-balanced occlusion than in the control group, which was contrary to our results. We speculated that disc remodeling may manifest different directions of volume changes under different pathological stages and in different species.

When we investigated the disc length based on the 3D model, we found length of the TMJ disc in the ADDwoR side was significantly shorter than that in the non-affected side. Amaral et al. classified disc morphology of ADDwoR disc into five types: thick posterior band, biconvex, biplanar, folded, and rounded, and all five types were shown as the decrease of the disc length. They also found that there were more severe deformations and higher prevalence of TMJ disc fold in ADDwoR patients compared with ADDwR patients [23], which was consistent with our findings. Cai et al. followed up 62 patients with disc displacement who had not received any treatment, and noted that the average disc length changed from 8.31 to 6.91 mm after 10.9 months follow-up, showing that the TMJ disc would likely become shorter during the natural course of disc displacement [24]. Therefore, the length of disc is a vital parameter for assess the severity of the ADDwoR.

Fifteen patients who experienced unilateral or bilateral disc repositioning surgery were included for assessing the three-dimensional morphological improvement of the disc following disc repositioning surgery. The volume, superficial area, length and width of TMJ disc were significantly increased after six months follow-up. These findings was consistent with that of Liu et al. who followed up 61 patients experienced disc repositioning surgery for more than 5 years, and observed that a longer disc length after surgery was correlated with a more harmonious disc-condyle relationship [15]. Although the disc mainly showed resorption after the disc displaced anteriorly, there was a tendency that volume superficial area, length and width of TMJ disc could recover to non-affected discs after disc repositioning surgery. We speculated that the results may be accounted for the normal relationship of disc-condyle achieved by surgery and the expansion of TMJ cavity created by the stabilized splint therapy after surgery. Therefore, the length of TMJ disc could not only be one of the indexes in assessing the severity of ADDwoR condition, but also act as an important parameter to evaluate surgical outcomes in these patients.

Previous studies had demonstrated the decrease of condyle height in patients with ADDwoR [25, 26], but the factors that may give rise to the results were unclear. Therefore, our study identified the correlation between the disc morphological changes and condylar height changes. We found that the degree of disc length decrease was significantly associated with the condylar height decrease. A previous study using finite element analysis demonstrated that disc displacement induced an increase of pressure in the disc and frictional coefficients on the condyle [27], thus a possible explanation for the correlation between condylar height and disc morphological changes could be that the condylar motion squeezes the disc forward, increases stress on the surfaces of condyle and disc, which results in degeneration, then the condyle height and disc length were decreased consequently.

There are several limitations in this study. Disc semiautomated segmentation in the 3D reconstruction process requires an experienced doctor to modify the disc boundary, whereas automated segmentation, which could decrease observer error, will be more precise in disc remodeling if the advanced method is developed. The facial asymmetry of patients was not taken into account in the study, which may affect the accuracy of condylar height measurement. Moreover, a relatively small sample size and difference between sex and ages of the patients could bring about study bias, and future studies including a larger number of patients are desirable to precisely assess influence factors in disc morphological changes in different ADDwoR patients. Furthermore, the six months follow-up may not be the end of disc remodeling, and a longer follow-up is needed in our future studies.

## Conclusions

This study demonstrated that the TMJ discs tended to be morphologically smaller in volume and shorter in length under ADDwoR status, and the condylar height loss of ADDwoR TMJ was correlated with these morphological changes of TMJ discs. Importantly, the ADDwoR discs tended to morphologically recover toward non-affected discs after 6 months follow-up following TMJ disc repositioning surgery. The results imply that TMJ disc position was tightly related to physiological status of disc and condyle, and disc recapture might be benefit for condyle and disc itself repair.

## Supplementary Information

**Additional file 1.** Raw data of TMJ discs of ADDwoR patients.

**Additional file 2.** Raw data of TMJ discs following disc repositioning surgery.

**Additional file 3.** Stl files of TMJ discs in all patients.

**Additional file 4.** The measurement method of condylar height.

## Data Availability

The datasets used and/or analyzed during the current study available from the corresponding author on reasonable request.
