# Supplementary material for: Morphological changes of TMJ disc in surgically treated ADDwoR patients: a retrospective study
Source: BMC Oral Health. 2022 Oct 1;22:432. doi: 10.1186/s12903-022-02469-8 (PMC9526344; doi:10.1186/s12903-022-02469-8)
Supplement: Supplementary file 4 — Additional file 4. The measurement method of condylar height. [file 12903_2022_2469_MOESM4_ESM.docx]

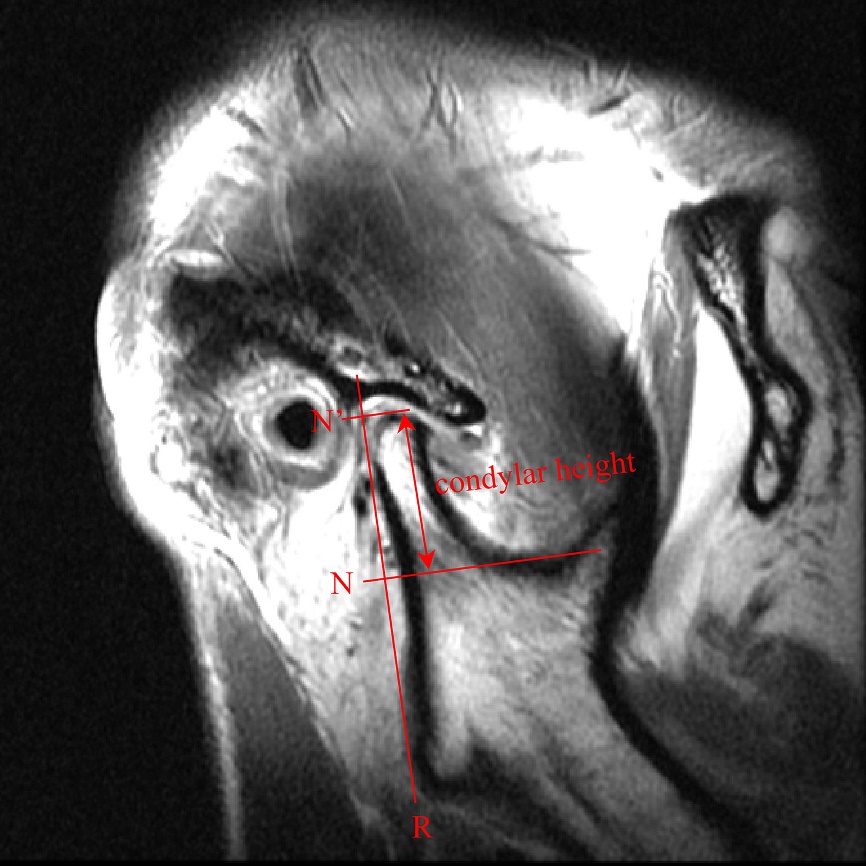


Supplemental Figure 1. The measurement of condylar height. Condylar height = The vertical distance between lines N and N'. Line R: the tangent of the posterior margin of mandibular ramus and the condyle. Line N: a vertical line of the line R and tangent to the lowest point of the mandibular sigmoid notch. Line N': a parallel to line N and tangent to the condylar head.
